# Mechanical correlates of dyspnea in bronchial asthma

**DOI:** 10.1002/phy2.166

**Published:** 2013-12-08

**Authors:** Andrea Antonelli, Emanuele Crimi, Alessandro Gobbi, Roberto Torchio, Carlo Gulotta, Raffaele Dellaca, Giorgio Scano, Vito Brusasco, Riccardo Pellegrino

**Affiliations:** 1 Allergologia e Fisiopatologia Respiratoria, ASO S. Croce e Carle, Cuneo, Italy; 2 Fisiopatologia Respiratoria, Dipartimento di Medicina Interna, Università di Genova, Genova, Italy; 3 TBM Lab, Dipartimento di Elettronica, Informazione e Bioingegneria, Politecnico di Milano, Milano, Italy; 4 Pneumologia‐Fisiopatologia Respiratoria, AOU S. Luigi Gonzaga, Orbassano (Torino), Italy; 5 Dipartimento di Medicina Interna, Sezione di Immunologia Clinica, Allergologia e Malattie Respiratorie, Università di Firenze, Firenze, Italy

**Keywords:** Airway mechanics, dyspnea descriptors, forced oscillation technique, lung hyperinflation, methacholine

## Abstract

We hypothesized that dyspnea and its descriptors, that is, chest tightness, inspiratory effort, unrewarded inspiration, and expiratory difficulty in asthma reflect different mechanisms of airflow obstruction and their perception varies with the severity of bronchoconstriction. Eighty‐three asthmatics were studied before and after inhalation of methacholine doses decreasing the 1‐sec forced expiratory volume by ~15% (mild bronchoconstriction) and ~25% (moderate bronchoconstriction). Symptoms were examined as a function of changes in lung mechanics. Dyspnea increased with the severity of obstruction, mostly because of inspiratory effort and chest tightness. At mild bronchoconstriction, multivariate analysis showed that dyspnea was related to the increase in inspiratory resistance at 5 Hz (*R*_5_) (*r*^2^ = 0.10, *P* = 0.004), chest tightness to the decrease in maximal flow at 40% of control forced vital capacity, and the increase in *R*_5_ at full lung inflation (*r*^2^ = 0.15, *P* = 0.006), inspiratory effort to the temporal variability in *R*_5‐19_ (*r*^2^ = 0.13, *P* = 0.003), and unrewarded inspiration to the recovery of *R*_5_ after deep breath (*r*^2^ = 0.07, *P* = 0.01). At moderate bronchoconstriction, multivariate analysis showed that dyspnea and inspiratory effort were related to the increase in temporal variability in inspiratory reactance at 5 Hz (*X*_5_) (*r*^2^ = 0.12, *P* = 0.04 and *r*^2^ = 0.18, *P* < 0.001, respectively), and unrewarded inspiration to the decrease in *X*_5_ at maximum lung inflation (*r*^2^ = 0.07, *P* = 0.04). We conclude that symptom perception is partly explained by indexes of airway narrowing and loss of bronchodilatation with deep breath at low levels of bronchoconstriction, but by markers of ventilation heterogeneity and lung volume recruitment when bronchoconstriction becomes more severe.

## Abstract (precis)

In asthma, respiratory symptom perception is qualitatively different depending on the level of bronchoconstriction. During the early phases of an attack dyspnea and its descriptors appear to be related to the severity of airway narrowing and loss of bronchodilatation with deep breath, whereas when narrowing worsens they depend on more profound changes in lung periphery as suggested by increased ventilation heterogeneity and reduced lung volume recruitment with breathing.

## Introduction

Dyspnea is one of the cardinal symptoms for asthma diagnosis, severity evaluation, and monitoring. Because of its impact on quality of life, physical activity, and choice of treatments, dyspnea has been the object of intensive research over the last few decades (Lougheed et al. 1993, 1995; Banzett et al. 2000; Killian et al. 2000; Lougheed 2007). Based on its relationships with changes in spirometry and lung volumes, the symptom is believed to arise from constricted airways or the inspiratory muscles working at high lung volume (Killian et al. 2000), where the elastic load is increased. The precise stimuli of dyspnea are, however, difficult to ascertain because (i) airway narrowing itself may be the trigger for lung hyperinflation (Pellegrino et al. 1993) and (ii) although breathing at high lung volumes is associated with an increased elastic work on inspiration, it tends to preserve airway patency, thus decreasing the total resistive work of breathing.

Dyspnea is generally reported by patients to indicate an uncomfortable stimulus often accompanied by multiple descriptors, the most common being chest tightness, difficulty of inspiration, unrewarded inspiration, and expiratory difficulty (Killian et al. 2000). In asthma, no association was found between chest tightness and work of breathing during induced bronchoconstriction (Binks et al. 2002). Thus, it is possible that different descriptors of dyspnea are related to different mechanical changes occurring during asthma attacks.

The bulk of studies on dyspnea and asthma used the forced expiratory volume in 1 sec (FEV_1_), which is insensitive to a series of mechanical changes associated with airway narrowing in response to constrictor stimuli. Among these are changes in the bronchodilator effects of the deep inspiration (DI) (Lim et al. 1987; Pellegrino et al. 1996) and the following velocity of renarrowing (Brusasco and Pellegrino 2003; An et al. 2007; Gobbi et al. 2013), development of serial and parallel ventilation heterogeneities (Pellegrino et al. 1996; Venegas et al. 2005a,b; Wanger et al. 2005; An et al. 2007; Winkler and Venegas 2011), and temporal variability in airway tone (Que et al. 2001; Frey et al. 2005; Gobbi et al. 2013). If respiratory symptoms are the result of specific and independent mechanisms (Banzett et al. 2000; Killian et al. 2000), then the above features may be differently associated with the descriptors of dyspnea. For instance, an increased stiffness of airway wall, which is likely associated with increased airway smooth muscle tone, could be reflected by an increased inspiratory effort to dilate the airways with a DI. Spatial ventilation heterogeneities and temporal airway instability could be a source of unrewarded inspiration, reflecting a difficulty to recruit closed or near closure airways.

On this ground, we tested the hypothesis that different descriptors of dyspnea reflect different mechanisms associated with airflow obstruction in asthma and their perception varies with the severity of airway narrowing. Overall dyspnea sensation and its descriptors were measured in mild asthmatics at baseline and after inhalation of methacholine (MCh), when their FEV_1_ was decreased by 10–20% of control (mild obstruction) and 20–30% of control (moderate obstruction). These conditions mimic the onset of a natural asthma attack and as such represent the first steps of an asthma attack. Symptoms were examined in relation to changes in respiratory mechanics assessed by a within‐breath forced oscillation technique (FOT) (Navajas and Farré 1999; Oostveen et al. 2003; Dellacà et al. 2004; LaPrad and Lutchen 2008) in addition to spirometry and lung volumes measurements.

## Material and Methods

### Subjects

Eighty‐three subjects with mild intermitted bronchial asthma (Global Initiative for Asthma 2007) were studied (Table 1). To be included, subjects had to be in stable clinical conditions, free from asthma exacerbations over the previous 4 weeks, without asthma treatments other than short‐acting bronchodilator on demand (Global Initiative for Asthma 2007). The study protocol was approved by the local Ethical Committee and written informed consent was obtained from each subject before entering the study.

**Table 1.** Subjects’ anthropometric characteristics and baseline lung function data.

Sex (m/f)	57/26
Age (years)	37 ± 12
Smoking habit, current/former/never	0/1/82
Height (cm)	172 ± 10
BMI (Kg·m^−2^)	24 ± 3
FEV_1_, % of predicted	94 ± 14
FEV_1_/FVC,%	75 ± 8
TLC, % of predicted	101 ± 10
FRC, % of predicted	96 ± 18
RV, % of predicted	103 ± 26

Data are mean ± SD. BMI, body mass index; FEV_1_, forced expiratory volume in 1 sec; FVC, forced expiratory vital capacity; TLC, total lung capacity; FRC, functional residual capacity; RV, residual volume.

### Lung function measurements

Spirometry, maximal flow–volume curves, and absolute lung volumes were obtained in a body plethysmograph (Autobox, SensorMedics Inc., CA) following the American Thoracic Society / European Respiratory Society (ATS/ERS) recommendations (Miller et al. 2005; Wanger et al. 2005). Briefly, thoracic gas volume was measured while subjects were panting against a closed shutter at a frequency slightly <1 Hz with their cheeks supported by hands. After the shutter was opened, the subjects took a full inspiratory capacity (IC) and then forcefully expired from total lung capacity (TLC) to residual volume (RV) for at least 6 sec to measure forced vital capacity (FVC) and 1 sec forced expiratory volume (FEV_1_). Functional residual capacity (FRC) was calculated from thoracic gas volume corrected for any difference between the volume at which the shutter was closed and the average end‐expiratory tidal volume of the four preceding regular breaths. Predicted values for spirometry and lung volumes were from Quanjer et al. (1993).

Partial forced expiratory maneuvers were recorded in the body plethysmograph (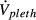) as follows. After at least four regular breaths, thoracic gas volume was measured while subjects were panting against a closed shutter at a frequency slightly <1 Hz with cheeks supported by hands. After the shutter was opened, the subjects were asked to perform a forced expiration to residual volume from about 70% of control vital capacity to RV. 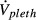 was measured at 40% of control FVC. Combined partial and maximal expiratory forced expiratory maneuvers were obtained in the body plethysmograph. After at least four regular breaths, thoracic gas volume was measured while subjects were panting against a closed shutter at a frequency slightly <1 Hz with cheeks supported by hands. After the shutter was opened, the subjects were asked to perform a forced expiration to RV from about 70% of control vital capacity to RV (partial maneuver). This was immediately followed by a full inspiration and, without any breath holding, by another forced expiration to RV (maximal maneuver) (Pellegrino et al. 1996, 1998). Flows were measured on the partial and maximal flows (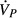 and 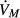, respectively) at 40% of control FVC.

Respiratory impedance was measured by a FOT system previously described (Gobbi et al. 2009, 2013). Sinusoidal pressure oscillations (5, 11, and 19 Hz frequency, ~ 2‐cm H_2_O amplitude) were generated by a 16‐cm‐diameter loudspeaker (model CW161N, Ciare, Italy) and applied at the mouth during tidal breathing. The loudspeaker was mounted in a rigid plastic box and connected in parallel to a mesh pneumotachograph and mouthpiece on one side and to a low‐resistance high‐inertance tube on the other side. Overall load under this breathing frequency (BF) was 0.98 cm H_2_O·sec·L^−1^. Airway opening pressure and flow were recorded by piezoresistive transducers (DCXL10DS and DCXL01DS; Sensortechnics, Germany, respectively) and sampled at 200 Hz. A 15 L/min bias flow of air generated by an air pump (CMP08, 3A Health Care, Italy) was used to reduce the dead space to about 35 mL. Respiratory resistance (*R*) and reactance (*X*) were computed by a least squares algorithm (Kaczka et al. 1995, 1999) at 5 Hz (*R*_5_ and *X*_5_, respectively) and 19 Hz (*R*_19_ and *X*_19_, respectively). Artifacts due to glottis closure or expiratory airflow limitation were avoided by discarding breaths showing any of the following features: (i) tidal volume <0.1 L or >2.0 L, (ii) difference between measured flow oscillation and ideal sine wave with the same Fourier coefficients >0.2 (Marchal et al. 2004), and iii) ratio of minimum to average *X* > 3.5 (Gobbi et al. 2009). The same breaths were used to measure tidal volume (*V*_T_), BF, and minute ventilation (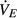).

### Symptom assessment

Dyspnea was defined as a general sense of discomfort perceived during tidal breathing (Killian et al. 2000); its main descriptors were chest tightness, inspiratory effort, unrewarded inspiration, and difficult expiration. A modified Borg scale was used to score the intensity of each descriptor with dyspnea being the sum of them. Special care was taken in the prestudy day to make sure that the subjects were fully informed of the aim of the study and familiarized with the definitions of the symptoms before the challenge so that they could properly rate the intensity of the descriptors.

### Study protocol

#### Prestudy day

After spirometry and lung volumes measurements, subjects underwent a standard inhalation challenge with the subjects inhaling doubling doses of MCh from 20 *μ*g during tidal breathing until the FEV_1_ was decreased by 20% or more from baseline. Dry powder MCh chloride (Laboratorio Farmaceutico Lofarma, Italy) dissolved into 3 mL of distilled water was aerosolized by an ampoule–dosimeter system (MB3 MEFAR, Brescia, Italy), delivering particles with a median mass diameter ranging between 1.53 and 1.61 *μ*m, and inhaled during spontaneous tidal breathing from FRC in a sitting position. The doses of MCh causing the FEV_1_ to decrease by 15% (PD_15_FEV_1_) and 25% (PD_25_FEV_1_) were calculated by interpolation of dose–response curves. Borg score was measured at each step. To enter the study, the subjects had to report a Borg score between 1 and 5 when the FEV_1_ was decreased by approximately 20% from control (Boulet et al. 1994).

#### Study day

The subjects attended the laboratory to undergo a modified MCh challenge using the predetermined PD_15_FEV_1_ and PD_25_FEV_1_ . At baseline, measurement included in order i) FOT applied during 7 min of tidal breathing with a DI taken at the end of the 5th min ii) Borg scores for dyspnea descriptors and oxygen saturation (SaO_2_) (SPIROPRO, Viasys Healthcare, Yorba Linda, CA) iii) three sets of partial forced expiratory maneuevers, and iv) three sets of combined partial and maximal maneuvers. MCh was delivered to the subjects with the tidal breathing method to avoid the effects of the deep breath on bronchial tone. Measurements began 2 min after the inhalation of the agent and proceeded in the same order of baseline. The only difference was that after MCh partial and combined partial and maximal flow–volume loops were recorded as single sets. The study was interrupted after the last predetermined dose or before if subjects asked for the test to be interrupted. The subjects were given aerosol albuterol for symptoms relief before dismissal.

### Data reduction and statistical analysis

The levels of bronchoconstriction were defined on the basis of the decrease in FEV_1_ (>10% and <20% for mild; and >20% and <30% for moderate).

*R*_5_, *R*_5‐19_ difference, and *X*_5_ recorded before DI were used to compute the interquartile ranges (IQR) of their probability density estimates (*R*_5_IQR_, *R*_5‐19_IQR_, and *X*_5___IQR_, respectively) and taken as estimates of short‐term temporal variability in bronchial tone. The difference *R*_5‐19_ was taken as an index of serial and parallel heterogeneities.

**Figure 1.**
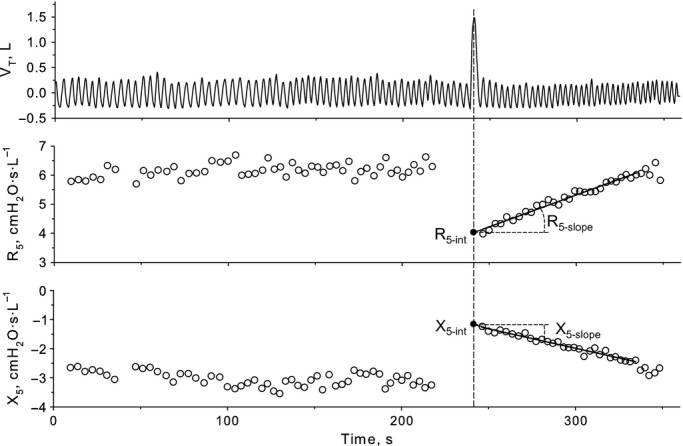
Tidal volume (V_T_), and inspiratory resistance (*R*_5_) and reactance (*X*_5_) measured at 5 Hz before and for 2 min after a deep inspiration (DI) during methacholine challenge in a typical subject. Circles are average values of *R*_5_ and *X*_5_ for each breath. The oblique lines represent the linear regression of values recorded after DI against time until pre‐DI values were reached. The intercept is the back‐extrapolated value at the time DI ended.

Values of *R*_5_ and *X*_5_ recorded after DI were submitted to linear regression analysis against time (Fig. 1). The regression intercepts at the time of full inflation (*R*_5‐int_ and *X*_5‐int_) were used, together with the ratio of 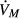 /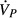, to assess the bronchodilator effect of volume history. The regression slopes (*R*_5‐slope_ and *X*_5‐slope_) were taken as estimates of velocity of airway renarrowing and reclosure, respectively.

A repeated measure analysis of variance (ANOVA) with Holm–Sidak multiple‐comparison test was used for statistical analysis of differences. Relationships between changes in lung function and symptoms were assessed by a forward stepwise regression analysis including as independent variables all parameters that were significantly correlated by univariate analysis (Pearson's simple correlation) with dyspnea or its descriptors. Values of *P* < 0.05 were considered statistically significant. Data are presented as mean ± standard deviation (SD).

## Results

The number of observations was of 83 at baseline, 64 at mild obstruction, and 63 at moderate obstruction. This was because not all MCh doses caused the decrease in FEV_1_ expected from prestudy day or the test was interrupted upon subjects’ request.

**Figure 2.**
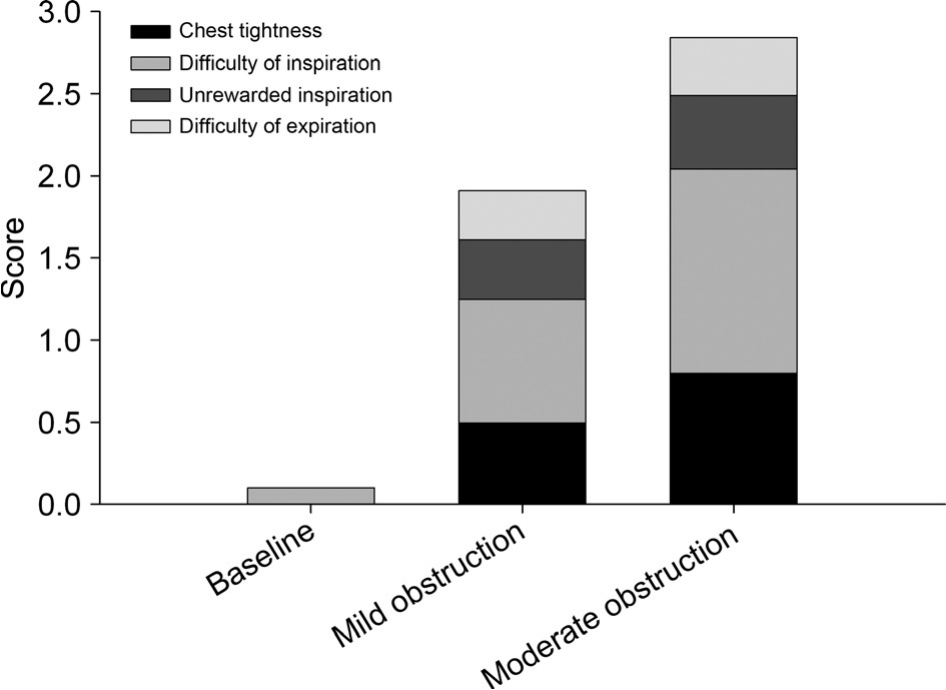
Dyspnea and its descriptors at baseline and at mild and moderate bronchoconstrictor levels.

Dyspnea increased significantly with the severity of obstruction, more because of inspiratory effort and chest tightness than unrewarded inspiration or expiratory difficulty (Fig. 2).

**Figure 3.**
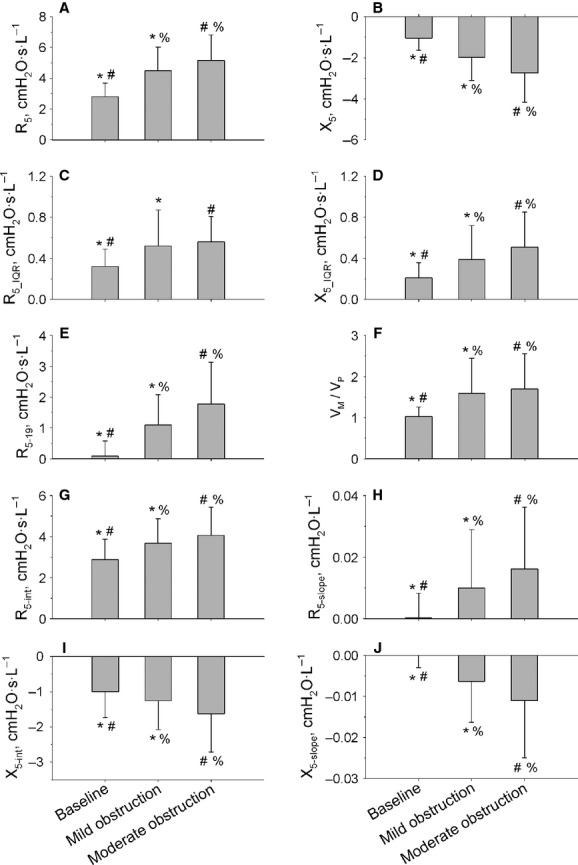
Main FOT parameters at baseline and at mild and moderate bronchoconstrictor levels. Panels (A, B) inspiratory resistance (*R*_5_) and reactance (*X*_5_) at 5 Hz. Panels (C, D) interquartile ranges of the probability density estimation of *R*_5_ (*R*_5___IQR_) and *X*_5_ (*X*_5___IQR_). Panel (E) difference inspiratory resistance between 5 and 19 Hz (*R*_5‐19_). Panel (F) ratio of maximal‐to‐partial forced expiratory flow at 40% control forced vital capacity (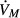/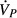). Panels (G, H), intercept (*R*_5‐int_), and slope (*R*_5‐slope_) of the linear regression analysis of the post‐DI *R*_5_ values over time. Panels (I, J), intercept (*X*_5‐int_), and slope (*X*_5‐slope_) of the linear regression of the post‐DI *X*_5_ values over time. Pairs of symbols indicate statistically significant differences between conditions.


As expected, the MCh‐induced reductions in FEV_1_ were paralleled by significant decrements in FVC, 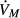, 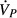, and 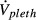 (Table 2). These changes were accompanied by increments of FRC and RV, indicating the occurrence of lung hyperinflation and gas trapping with the constrictor agent. *R*_5_ also increased, while *R*_5_–*R*_19_ increased and *X*_5_ became more negative, suggesting an increase in ventilation heterogeneity associated with bronchoconstriction. Moreover, both *R*_5_IQR_ and *X*_5___IQR_ increased, suggesting increased temporal fluctuations of airway narrowing and closure. The 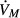/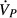 ratio and *R*_5‐int_ increased during bronchoconstriction, whereas *X*_5‐int_ decreased, suggesting partial bronchodilatation and recruitment of lung volume with the DI. *R*_5‐slope_ and *X*_5‐slope_ increased, suggesting faster airway renarrowing and closure. These results are shown in Figure 3.

**Table 2.** Main lung function parameters before and after methacholine.

	Baseline	Mild obstruction (FEV_1_, 15 ± 2% decrease)	Moderate obstruction (FEV_1_, 25 ± 3% decrease)
FEV_1_ (L)	3.45 ± 0.81*^§^	2.96 ± 0.68*^#^	2.57 ± 0.61^§^^#^
FVC (L)	4.58 ± 0.96*^§^	4.29 ± 0.86*^#^	3.95 ± 0.88^§^^#^
TLC (L)	6.42 ± 1.17	6.46 ± 1.16	6.31 ± 1.17
FRC (L)	3.03 ± 0.74*^§^	3.33 ± 0.75*^#^	3.43 ± 0.70^§^^#^
RV (L)	1.84 ± 0.55*^§^	2.16 ± 0.65*^#^	2.34 ± 0.61^§^^#^
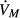 , (L sec^−1^)	2.43 ± 1.08*^§^	1.44 ± 0.73*^#^	0.95 ± 0.50^§^^#^
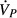 , (L sec^−1^)	2.39 ± 1.02*^§^	1.07 ± 0.60*^#^	0.69 ± 0.49^§^^#^
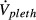 , (L sec^−1^)	3.43 ± 1.28*^§^	1.50 ± 0.86*^#^	1.09 ± 0.71^§^^#^
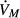 / 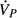 , units	1.03 ± 0.23*^§^	1.59 ± 0.86*^#^	1.70 ± 0.87^§^^#^
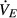 , (L·min^−1^)	13.3 ± 4.0*	12.4 ± 4.7*^#^	13.1 ± 4.7^#^
BF (min^−1^)	14 ± 4*^§^	15 ± 4*^#^	16 ± 5^§^^#^
V_T_ (L)	1.1 ± 0.4*^§^	0.9 ± 0.30*	0.9 ± 0.4^§^
SaO_2_, %	97.2 ± 1.3*^§^	96.8 ± 1.3*	96.7 ± 1.4^§^

Data are mean ± SD. 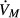 (maximal) and 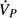 (partial) forced expiratory flows at 40% of control FVC; 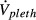, plethysmographic partial forced expiratory flow at 40% of control FVC; 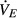, minute ventilation; BF, breathing frequency; V_T_, tidal volume, SaO_2_, oxygen saturation. BMI, body mass index; FEV_1_, forced expiratory volume in 1 sec; FVC, forced expiratory vital capacity; TLC, total lung capacity; FRC, functional residual capacity; RV, residual volume. Pairs of symbols indicate statistically significant differences between conditions.

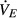
 remained stable during the challenge, even though BF was slightly but significantly increased and V_T_ decreased (Table 2). SaO_2_ decreased significantly, but minimally with MCh (Table 2). Intensity and quality of dyspnea were rated similarly between males and females (Table 4).

### Relationship between dyspnea sensation and lung function

At the level of mild bronchoconstriction, the following simple correlations between symptoms and absolute or percent changes in lung function were found to be significant: dyspnea versus *R*_5_ (*r* = 0.34; *P* = 0.008) and *R*_5‐19___IQR_ (*r* = 0.29; *P* = 0.022); chest tightness versus 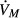 (*r* = −0.33; *P* = 0.009), R_5‐int_ (*r* = 0.29; *P* = 0.024), and 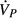 (*r* = −0.26; *P* = 0.040); inspiratory effort versus 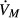 (*r* = −0.31; *P* = 0.013), and *R*_5‐19___IQR_ (*r* = 0.38; *P* = 0.003); and unrewarded inspiration versus *R*_5‐slope_ (*r* = 0.29; *P* = 0.027). Expiratory effort was not significantly related to any mechanical change. By multivariate analysis (Table 3), dyspnea remained significantly related to percent increase in *R*_5_ only (*r*^2^ = 0.10; *P* = 0.004), chest tightness to percent decrease in 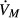, and percent increase in *R*_5‐int_ (*r*^2^ = 0.15; *P* = 0.006), and inspiratory effort to *R*_5‐19___IQR._ (*r*^2^ = 0.13; *P* = 0.003).

**Table 3.** Explanatory models for symptom variability with methacholine.

	Mild obstruction	Moderate obstruction
Dyspnea	R_5_ (*r*^2^ = 0.10)	X_5___IQR_ (*r*^2^ = 0.12)
Chest tightness	R_5‐int_ + 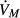 (*r*^2^ = 0.15)	None
Inspiratory effort	R_5‐19_IQR_ (*r*^2^ = 0.13)	X_5___IQR_ (*r*^2^ = 0.18)
Unrewarded inspiration	R_5‐slope_ (*r*^2^ = 0.07)	X_5‐int_ (*r*^2^ = 0.07)
Expiratory difficulty	None	None

*R*_5_ and *X*_5_, respiratory resistance and reactance at 5 Hz, respectively; *R*_5‐19_, difference between *R* at 5 and 19 Hz; *R*_5‐int_, *X*_5‐int_, and *R*_5‐slope_, intercepts and slope of the linear regression analysis of the postdeep inspiration *R*_5_ and *X*_5_ plotted versus time (see also Fig. 1); *R*_5‐19_IQR_ and *X*_5___IQR_, interquartile ranges of the probability density estimation of *R*_5‐19_ and *X*_5_.

At the level of moderate bronchoconstriction, the following simple correlations between symptoms and absolute or percent changes in lung function were found to be significant: dyspnea versus *R*_5_ (*r* = 0.25; *P* = 0.051), *R*_5_IQR_ (*r* = 0.26; *P* = 0.047), *X*_5___IQR_ (*r* = 0.36; *P* = 0.005), and *R*_5‐19_IQR_ (*r* = 0.35; *P* = 0.006); inspiratory effort versus *X*_5___IQR_ (*r* = 0.44; *P* < 0.001) and *R*_5‐19_IQR_ (*r* = 0.36; *P* = 0.005); and unrewarded inspiration versus *X*_5‐int_ (*r* = 0.30; *P* = 0.036). Neither chest tightness nor expiratory effort was correlated with any mechanical changes. By multivariate analysis (Table 3), dyspnea and inspiratory effort remained significantly related to absolute increase in *X*_5___IQR_ only (*r*^2^ = 0.12, *P* = 0.04 and *r*^2^ = 0.18, *P* < 0.001, respectively), and unrewarded inspiration to *X*_5‐int_ (*r*^2^ = 0.07, *P* = 0.04). No significant correlations were found between the increase in FRC and dyspnea or its descriptors at either levels of bronchoconstriction.

## Discussion

The main results of this study are that symptom perception during a MCh challenge was partly explained by functional parameters reflecting airway narrowing and loss of ability to dilate airways by DI at low level of bronchoconstriction, and ventilation heterogeneity at moderate level of bronchoconstriction.

### Results from previous studies

In asthma, dyspnea is believed to signal the severity of airflow obstruction (Banzett et al. 2000; Killian et al. 2000). Previous studies came to the conclusion that the diversity in dyspnea perception reflects different stimuli and pathways. For instance, chest tightness is thought to originate from stimulation of pulmonary irritant receptors (Killian et al. 2000; Filippelli et al. 2003; Parshall et al. 2012). Given the sensitivity of these receptors to a multiplicity of stimuli (Coleridge and Coleridge 1986), the trigger for chest tightness in asthma could be either chemical or mechanical. Inspiratory effort is another symptom frequently reported by asthmatics and is believed to originate from an increase in motor command to inspiratory muscles working at increased lung volumes because of dynamic hyperinflation. Several pieces of evidence appear to corroborate this notion. The increase in FRC has been shown to account for most of the increase in dyspnea (Lougheed et al. 1993, 1995; Filippelli et al. 2003; Lougheed 2007), and this was apparently the result of an increased inspiratory threshold load (Lougheed et al. 1995). Moreover, Lougheed and O'Donnell (Lougheed 2007) found that dyspnea and lung hyperinflation increased during induced bronchoconstriction even when the FEV_1_ had reached a plateau. Unrewarded inspiration has also been reported in asthma, presumably as a result of hypercapnia and hypoxia (Killian et al. 2000; Parshall et al. 2012), though this has not been proven (Lougheed et al. 1995). Expiratory difficulty has been usually neglected in previous studies (Laveneziana et al. 2006; Lougheed 2007) and the underlying physiological mechanisms never thoroughly examined.

### Comments on methodology

In comparison with previous studies, the present one has the strength that it was not limited to standard pulmonary function tests, but included FOT. This technique is sensitive to serial and parallel mechanical inhomogeneities, presumably occurring at the level of peripheral airways (DuBois et al. 1956; Lutchen and Gillis 1997; Gillis and Lutchen 1999; Downie et al. 2013), and allows to measure rapid changes in lung function within or between tidal breaths, such as those occurring during and after a DI (Navajas and Farré 1999; Black et al. 2003, 2004; Dellacà et al. 2004; Brown et al. 2007; LaPrad and Lutchen 2008; Gobbi et al. 2013).

We acknowledge some limitations of our study. First, it was conducted in a laboratory setting and not in real life. Although this does not invalidate the data because both quality and intensity of dyspnea, wheezing and cough in real life conditions are quite well reproduced during a bronchial challenge (Banzett et al. 2000; Lévesque et al. 2010), a possibility remains that different relationships between lung function and symptoms exist during severe natural asthma attacks or chronic airway narrowing. Further investigations are needed however, to validate the assumption that respiratory symptoms in a laboratory setting reflect those in real life. Second, the relationships between symptoms and lung function were examined by regression analysis, which does not necessarily imply a causal relationship between variables. Therefore, our findings need to be interpreted with caution. Third, the study was designed to evaluate interindividual differences in symptom perception over narrow ranges of bronchoconstriction. Thus, psychological and emotional differences between subjects likely represented the major source of unexplained variability. Fourth, selection of the subjects was limited to mild‐intermittent asthmatics to avoid the effects of sensory adaptation to the chronic asthmatic condition. Finally, airway inflammation could contribute to symptoms (Sont et al. 1995), but was out of the purpose of this study.

### Interpretation of results

As expected, dyspnea increased linearly with the reduction in FEV_1_, confirming that airflow obstruction is an important determinant of symptoms in asthma. What this study adds is that changes in respiratory mechanics other than change in FEV_1_ contribute differently to dyspnea depending on the severity of airway narrowing.

At mild level of induced bronchoconstriction, that is, when the FEV_1_ was similarly decreased by approximately 15% in all subjects, functional predictors of symptoms were the increase in *R*_5_ for dyspnea, increase in *R*_5‐int_ and decrease in 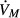 for chest tightness, short‐term variability in *R*_5‐19_IQR_ for inspiratory effort, and increase in *R*_5‐slope_ for unrewarded inspiration. *R*_5_ is determined not only by airway caliber of both large and small airways but also by the viscoelastic properties of the respiratory system and perhaps ventilation heterogeneity. Assuming that the viscoelastic properties of chest wall were not substantially affected by MCh, the additional contribution of *R*_5_ to dyspnea at a given decrease in FEV_1_ could be explained by changes within the lung to which FEV_1_ is insensitive. Chest tightness was significantly related to *R*_5‐int_, which is a measure of the bronchodilator effect of DI, with larger values indicating an impaired ability to distend constricted airways (Fig. 1). Thus, the significant relationship between chest tightness and *R*_5‐int_ could be the result of signals originating from contracted airways hard to distend by increase in lung volume. This finding is in line with a previous study showing that part of dyspnea was somewhat linked to the inability to distend the airways with a DI, as assessed by the maximal‐to‐partial forced expiratory flow ratio (Sont et al. 1995). Dyspnea and inspiratory effort correlated with *R*_5‐19___IQR_. Modeling studies have suggested that the frequency dependence of respiratory resistance is a reflection of heterogeneous ventilation at the level of peripheral airways (DuBois et al. 1956; Lutchen and Gillis 1997; Gillis and Lutchen 1999). However, Downie et al. (2013) recently found no correlation between *R*_5‐19_ and indexes of heterogeneous ventilation by nitrogen multibreath washout and suggested that this was due to lack of sensitivity to peripheral heterogeneity at frequencies ≥5 Hz. *R*_5‐slope_ is an index of airway renarrowing after a DI, which has been shown to be increased in asthmatic compared with healthy subjects (Black et al. 2003; Gobbi et al. 2013), presumably reflecting an increased velocity of airway smooth muscle shortening (Stephens et al. 2003; Bullimore et al. 2011). Thus, the significant relationship between unrewarded inspiration and *R*_5‐slope_ may reflect the lack of the persisting bronchodilatation a subject would expect to experience after a DI. Whatever the reasons for associations between changes in lung mechanics and specific descriptors of dyspnea, these results suggest that temporal variability, stiffness, and shortening velocity of airway smooth muscle in conducting airways already contribute to signal the asthma attack since the early stages.

At moderate level of induced bronchoconstriction, that is, when the FEV_1_ was decreased by approximately 25% in all subjects, predictors of symptoms were the increase in *X*_5___IQR_ for both dyspnea and inspiratory effort, and the increase in *X*_5‐int_ for unrewarded inspiration. Recent reports indicate that temporal variability in bronchial tone in asthma is associated with an increased risk of severe asthma episodes (Frey et al. 2005; Gulotta et al. 2012), presumably because fluctuations in biological signals often follow power law distributions and hence carry more useful information than the mean values (Frey and Suki 2008). Imaging and modeling studies support the idea that severe bronchoconstriction might be the result of clusters of poorly ventilated lung regions forming when the constrictor response of the peripheral airways is very heterogeneous and associated with central airway narrowing (Venegas et al. 2005a,b; Winkler and Venegas 2011). To the extent that *X*_5___IQR_ reflect the heterogeneous distribution of ventilation within the periphery of the lung (LaPrad and Lutchen 2008; Downie et al. 2013), this study would suggest that this mechanism has the potential to contribute to dyspnea and inspiration effort when bronchoconstriction becomes more severe. In this context, the relationship between increase in *X*_5‐int_ and unrewarded inspiration would suggest that if a large breath (DI) is not sufficient to recruit closed or near closure airways (*X*_5_), thus sufficiently rewarding the sense of breathing, then a much smaller breath such as a tidal breath will be even less effective to the aim. Altogether, these results indicate that temporal variability in peripheral airway closure and inability to recruit poorly ventilated regions may contribute to symptoms associated with moderate bronchoconstriction in asthma. According to current knowledge, dyspnea in asthma is caused by stimuli arising from the irritant receptors and bronchial C fibers reaching the central nervous system via vagus nerve (Coleridge and Coleridge 1986; Banzett et al. 2000). With bronchoconstriction, rapidly adapting stretch receptors are stimulated by chemicals, airway narrowing itself, and local flow, whereas bronchial C fibers mostly by chemicals (Coleridge and Coleridge 1986). Thus, the relationships of inspiratory effort to the increase in *R*_5‐19_IQR_ at mild bronchoconstriction, and *X*_5___IQR_ at moderate bronchoconstriction, may suggest that ventilation shifts from poorly ventilated regions where airway control is quite unstable over time to better ventilated regions. This would evoke large responses from the irritant receptors exposed to the increased flow (Coleridge and Coleridge 1986). A role of irritant receptors is also suggested by the shift to a more rapid and shallow breathing after MCh (Coleridge and Coleridge 1986). If so, then the resulting increase in inspiratory effort could reflect the difficulty to accommodate ventilation within constrained lung volumes and overactivation of irritant receptors.

In no instance the increase in FRC contributed to dyspnea or its descriptors. This appears to be in net contrast with studies documenting significant correlations between lung hyperinflation and breathlessness in asthmatics exposed to MCh (Lougheed et al. 1993, 1995; Filippelli et al. 2003; Lougheed 2007). Although in two of these studies the increase in FRC was much larger than in our subjects, namely, 1.35 L versus 0.4 L (Lougheed et al. 1993, 1995), thus potentially explaining that it takes quite large increments in lung volumes to evoke the symptom, this was not the case of the two other studies (Filippelli et al. 2003; Lougheed 2007), where the increase in the FRC was much smaller, namely, 0.62 L to 0.55 L. Apart from the differences in design, methodology, and data analysis between this and the above studies, which may explain in part the different results, we believe that at levels of airflow obstruction and lung hyperinflation as in this study the mechanical events occurring within the airways represent a primary source of neural signals evoking symptoms. Only with the increase in motor output to overcome the elastic work of breathing signals from the chest wall presumably contribute to breathlessness. However, it must also be considered the case that, with the increase in FRC, the airways tend to dilate, thus blunting the severity of airway narrowing and the expected increase in symptoms as a result of lung hyperinflation.

The fall in FEV_1_ was not correlated with the increase in dyspnea or any of its descriptors either at mild or moderate levels of bronchoconstriction. This cannot be ascribed to a low quality of the measurements, which fully satisfied the requirements of the ATS/ERS for lung function testing (Miller et al. 2005; Wanger et al. 2005), or can be explained by inclusion of poor symptom perceivers because subjects were selected among those with a Borg of score between 1 and 5 at a decrease in FEV_1_ by 20% from on the prestudy MCh challenge (Boulet et al. 1994). Previous studies reported controversial results in this respect (Lougheed et al. 1993; Sont et al. 1995), with only modest correlation coefficients in some cases (Killian et al. 2000; Filippelli et al. 2003). In this study, correlations between lung function variables and symptoms were analyzed at nearly fixed decrements of FEV_1_. Thus, the variability in FEV_1_ within levels of bronchoconstriction was probably too narrow to yield significant correlations.

Despite the sophisticated methodology used in this study, the variance in symptoms explained by changes in lung function never exceeded 24%, which is in line with previous studies. This is likely because symptoms are signaled by a broad series of sensors strategically placed from the upper airways to the lungs, within the cardiovascular system, and in respiratory muscles. It is thus clear that lung mechanics are just one of the many rings of a long chain and as such cannot contribute to the symptom more than in a minor part. Emotion and affection are two major dynamic dimensions that importantly modulate or amplify the perception of the respiratory sensation. Finally, we report that expiratory difficulty was not related to any functional change. One possible reason is that the level of bronchoconstriction was too low and hardly reported by the patients.

SaO_2_ was slightly decreased after MCh. This is unlikely to have contributed to our results because hypoxia has been shown to decrease the perception of load (Eckert et al. 2005), which is the opposite to the increase in symptoms after MCh. Finally, our results are at variance with those of Killian et al. (2000), who reported relationships between dyspnea or its descriptors and main anthropometric characteristics, or baseline lung function, or degree of airway responsiveness. This might have been due to insufficient power of the present study in this respect, though it must be noted that in the Killian's study (Killian et al. 2000) the contribution of baseline lung function, degree of airway responsiveness, age, height, and weight represented a minor contribution to dyspnea and its descriptors (Lougheed 2007). Similarly to Lougheed et al (2006), we did not find significant differences in dyspnea or any descriptors during the challenge as a function of gender. Others found that women perceive the intensity symptoms more than men (Killian et al. 2000) or the disease itself rather than the symptoms (Nowobilski et al. 1997). Taken together, these data suggest that the differences are not presumably large and consistent and may be revealed only when large numbers of patients are studied.

## Conclusions

To our knowledge, this is the first study showing that symptom perception in asthma is qualitatively different depending on the level of bronchoconstriction. In particular, dyspnea seems to reflect a difficulty in redistributing inspiratory flow and dilate airways with DI. In addition, our data suggest that symptoms may reflect changes in lung mechanics in more central airways during early phases of an asthma attack and in more peripheral airways when bronchoconstriction becomes more severe. However, because only a part of symptom variability was explained by changes in lung mechanics, quantitative and qualitative assessment of dyspnea cannot provide information on the severity of the underlying functional abnormalities. In this context, simple measurement of pulmonary impedance by FOT may result as useful to detect ventilation defects, which are regarded as responsible for severity of the disease.

## Acknowledgments

We are grateful to Fondazione Giovanni e Annamaria Cottino (Turin, Italy) for financial support, and to SOL (Monza, Italy) for technical assistance.

## Conflict of Interest

AG, RD and Politecnico di Milano University (Institution of AG and RD) own stocks of a spin‐off company of the Politecnico di Milano involved in the development of Forced Oscillation devices.
